# Quality of Clinical Notes Created by Ambient Listening Generative AI: Pragmatic Prospective Pilot Study

**DOI:** 10.2196/86474

**Published:** 2026-04-17

**Authors:** Sandra L Taylor, Melissa Jost, Scott MacDonald, Yunyi Ren, Shelley Hilton, Sadie Davenport, Debbie Aizenberg, Bruce Hall, Courtney R Lyles, Jason Y Adams

**Affiliations:** 1 Department of Public Health Sciences, School of Medicine, University of California, Davis, 4480 2nd Avenue, Suite 4152, Sacramento, CA, 95817, United States, 1 916-734-4800; 2 Clinical and Translational Science Center, University of California, Davis, Sacramento, CA, United States; 3 Department of Clinical Informatics, University of California, Davis, Sacramento, CA, United States; 4 IT Clinical Applications, University of California, Davis, Sacramento, CA, United States; 5 Blaisdell Medical Library, University of California, Davis, Sacramento, CA, United States; 6 Department of Otolaryngology, Head and Neck Surgery, School of Medicine, University of California, Davis, Sacramento, CA, United States; 7 Department of Internal Medicine, School of Medicine, University of California, Davis, Sacramento, CA, United States; 8 IT Enterprise Analytics and Data Services, University of California, Davis, Sacramento, CA, United States

**Keywords:** generative artificial intelligence, generative AI, artificial intelligence scribe, AI scribe, clinical documentation, ambient listening, quality of care

## Abstract

**Background:**

Physicians routinely document specifics of patient encounters in clinic visit notes, a critical but potentially time-consuming task. Ambient listening artificial intelligence (AI) technology is being integrated into clinical workflows to reduce documentation burden by creating draft visit notes. While this technology is promising, it is not perfect, and the potential for patient harm needs to be understood and mitigated. We developed and piloted an efficient, standardized approach to evaluating AI-generated notes for safety concerns in ambulatory care visits.

**Objective:**

The objective of this quality improvement project was to develop and pilot an efficient, standardized, and scalable approach to evaluating AI-generated notes for safety concerns in ambulatory care visits.

**Methods:**

During a 2-month pilot (July to August 2024), 31 physicians across multiple specialties used an ambient listening AI scribe to assist with the creation of 7545 clinic notes. A novel survey instrument was developed to assess note quality, focusing on 4 error types: accidental inclusions, accidental omissions, hallucinations, and bias. Physicians evaluated 356 (4.7%) AI-generated notes. Where an error was present, physicians rated its severity based on its potential to cause patient harm if it was not corrected, on a 0 to 5 scale. Additionally, a vendor-reported metric on the percentage of note content edited by physicians was analyzed.

**Results:**

Of the 356 evaluated notes, accidental omissions were the most frequent error (n=64, 18%), followed by hallucinations (n=41, 11.5%), and accidental inclusions (n=33, 9.3%). Bias was rare (n=4, 1.1%). Most (119/142, 83.8%) errors were rated as mild to moderate (severity 1‐3), with only 19 (5.3%) notes containing errors rated as posing serious or imminent risk (severity 4‐5). Editing metrics across all AI-created notes showed a median of 9.0% (IQR 2.5%-21.9%) of AI-generated words were changed, with 14.9% (143/960) of notes left entirely unedited. Physician editing practices varied widely, with average percentages of AI-generated words changed ranging from 1.9% to 69.3% (median 9.0%, IQR 2.5%-21.9%).

**Conclusions:**

AI-generated clinical notes were generally of high quality, with 94.7% (337/356) free from significant errors. However, because a small number contained errors that carried the risk of serious harm if not corrected, careful clinician review of notes remains imperative. Prior to deploying an AI scribe, organizations should pilot the technology and include an efficient review process to understand the nature and type of errors common at their organization. This pilot provides a scalable model for other health systems seeking to implement AI scribe technology responsibly.

## Introduction

Generative artificial intelligence (AI) is infiltrating and transforming many aspects of medicine, with specific emphasis on clinician-based tasks such as note creation and after-visit summaries, drafting responses to patient messages, and summarizing patient charts—all of which could reduce the clinical workload [1]. The specific uptake of AI scribes for note creation has some of the highest rates of implementation in the United States. These AI scribes use ambient listening generative AI technology to generate draft notes [2,3], capturing patient-physician conversations during encounters and converting them to documentation that is reviewed, modified, and approved by the physician. To date, the literature related to AI scribes has signaled improvements in time per note for physicians, with a potential for reducing workload and burnout [4,5].

While capitalizing on the potential benefits of AI scribes, the potential for patient harm also needs to be understood and mitigated. Generative AI is not perfect, and errors embedded in a patient’s medical record could impact care [6]. Indeed, concerns have been raised about the use of generative AI to create physician responses to patient inquiries [7], and more broadly, there is a need for rigorous validation of large language models in clinical practice [8]. Real-world validation of generative AI is essential to avoiding patient harm [4], but efficient assessment tools that capture the most critical errors are not yet widely available.

Therefore, as part of a quality improvement pilot program using an ambient listening AI scribe to assist in preparing clinic visit notes, the University of California, Davis Health (UCDH) developed a novel survey instrument to assess the quality of AI-generated notes and identify errors with potential to impact patient care and outcomes. The overall goal of the quality assessment was to ensure patient safety prior to widespread deployment of the AI scribe, with the following specific objectives: develop an efficient, scalable, and standardized approach to assess the quality of AI scribe–generated clinical visit notes; identify the type and frequency of errors introduced by the AI scribe; quantify the severity of different errors and potential for patient harm, and develop a long-term monitoring approach.

## Methods

### Study Design

The UCDH system is an academic medical center with a 653-bed teaching hospital and the only level I adult and pediatric trauma center in inland Northern California, serving a 33-county region of about 6 million residents with more than 1.9 million patient encounters annually. UCDH conducted an ambulatory care pilot program evaluating use of an AI scribe tool to assist with preparation of clinic visit notes. The pilot program ran for 2 months (July to August 2024), during which the physicians in the pilot had the option of using the ambient listening tool. We first summarized the total number of notes created by the AI scribe technology during the pilot period, overall and by physician, followed by a secondary subanalysis of AI scribe note quality.

### Ethical Considerations

This study was determined by the University of California, Davis institutional review board to not be human subjects research and therefore was exempt from review (2367684‐1). Because this study was exempt from review and patient information was aggregated, study-specific informed consent and privacy protections were not necessary. Physicians were not compensated for participating in the pilot program.

### Note Quality Assessment

All physicians in the pilot agreed to assess the quality of draft notes produced by the AI scribe technology using a novel standardized assessment instrument for a subset of their clinic encounters. To minimize assessment burden, physicians were asked to evaluate 10 draft notes on 2 different days during a 3-week period. This number was selected to represent most notes in their outpatient practice on these selected days, and each clinician received regular reminders during the pilot to complete their assessments. Note types included history and physical notes and progress notes; note type was not captured as part of the assessment.

Detailed quality ratings by physicians in the pilot were captured using a novel standardized assessment instrument accessible via an online Qualtrics (Qualtrics, LLC) survey (Figure S1 in Multimedia Appendix 1). The survey was developed in collaboration with our institution’s AI Oversight Committee and included four components of quality deemed most relevant to patient safety, specifically whether the AI scribe note had the following: (1) accidental inclusions, (2) accidental omissions, (3) hallucinations, or (4) bias in the draft language (Textbox 1). These components were informed by the Physician Documentation Quality Instrument (PDQI-9) and known deficiencies in AI and large language model summarization tools [9,10]. On the survey, physicians reported if any of these types of errors were present. If so, the physician rated the severity of the error on a scale of 0 to 5, with 0 representing negligible risk to the patient, physician, or health system and 5 representing potential for serious and imminent risk of harm. Within this guidance, severity ratings were based on individual physicians’ clinical judgment in the context of the visit. Table 1 provides examples of errors reported for each severity rating level.

Textbox 1. **Components of note quality assessment.**

**Accidental inclusion**

- Does the draft note contain inaccurate, real information discussed in the visit that was accidentally included or misattributed by the AI?

**Accidental omission**

- Does the draft omit information that you would have included in the note if you had written/dictated it without the AI?

**Hallucination**

- Does the draft note contain inaccurate, undiscussed, hallucinated information that was made up by the AI?

**Bias**

- Does the note appear to either include or omit information that might increase the risk that vulnerable populations/protected classes of patients are treated unfairly?

**Table 1.** Examples of errors in artificial intelligence (AI)–generated notes at each severity rating level.

Severity rating	Examples of AI documentation mistakes
0	Misattributed a medication dosage statement to the parent, although it was part of the clinician’s discussion of risks and benefits Inserted advice on substance abuse that the clinician did not discuss
1	Included irrelevant details (eg, ability to use computer) Attributed a medication side effect comment to the patient when it was stated by the clinician Missed noting supplement use and risk-benefit discussion Missed noting spine clinic appointment (instead wrote referral would be placed)
2	Electrocardiogram results reported as echocardiogram results Incorrect timeline for COVID-19 exposure Ovarian adenoma incorrectly documented rather than ovarian thecoma or fibroma Medication instructions simplified incorrectly (“take for one month” instead of conditional continuation) Anxiety medication documented for the patient instead of the spouse Misinterpretation of recommended blood test timing (“have blood drawn in 1 week” instead of 1 week before follow-up)
3	Included history of osteoporosis without supporting evidence Attributed discontinuation of a medication stopped years ago to one stopped yesterday Incorrectly stated that the patient was on methadone Missed discussion on home safety and assisted living recommendation Added the phrase “increase dose if symptoms improve,” which was contrary to the documented treatment plan
4	Incorrectly added directive to start aspirin for ankle pain, which was not the proposed plan Incorrectly stated that the patient has diabetes Did not include the patient’s discussion of weight loss, fatigue, and medication change Improper medication dose documented in plan
5	Attributed a history provided by the patient to an incorrect diagnosis Incorrectly stated that the patient should continue current metformin dosing Did not document computed tomography scan discussed by the clinician

Using these surveys, we summarized the proportion of each error type during the pilot. Furthermore, we calculated a quality score as follows. For each component of quality, a score ranging from 0 to 1 was calculated as 1 – (0.2 × severity) such that higher severity errors resulted in higher point deductions. The overall quality score was then calculated as the sum of the 4 component scores, yielding a score ranging from 0 (lowest quality) to 4 (highest quality). We also linked these quality ratings to patient characteristics for each note to report on the patient distribution in the sample.

The time between the clinic visit and the quality assessment review was calculated as the difference between the time that the AI scribe recording ended and when the physician started to review the note. Data for the quality assessment were collected separately from the AI scribe recording time data, which came from the vendor. We were not able to link the quality assessments and AI scribe data for 9 visits, and recording times were not available for 4 of the assessed notes.

For each note, the vendor calculated a measure of the percentage of the note that was retained, calculated as 1 – (number of word-level edits / number of words in the final completed note). Word-level edits are defined as additions, substitutions, and deletions. The vendor provided the average percentage retained for each physician during the pilot period. Due to technical issues, this measure could only be obtained at the note level from the vendor for 960 notes. We converted the percentage retained metric to the percentage of words changed.

## Results

A total of 31 physicians volunteered for the pilot program. Specialties of physicians in the pilot program included family practice (n=12, 38.7%); internal medicine (n=11, 35.5%); otolaryngology (n=3, 9.7%); pediatrics (n=2, 6.5%); and dermatology, obstetrics and gynecology, and endocrinology (n=1 each, 3.2%).

### Quality Assessment

**Figure 1.**
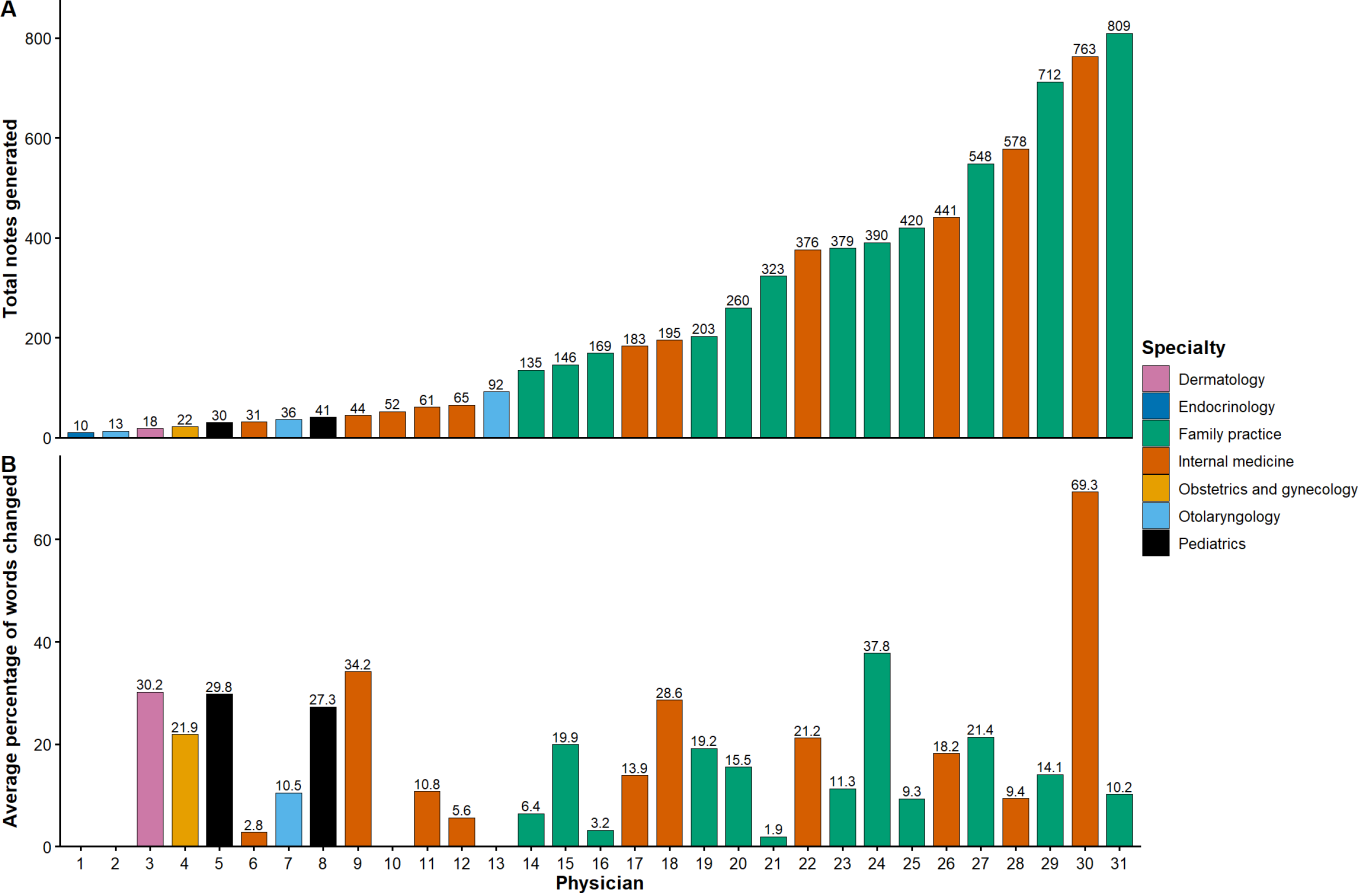
(A) Total number of notes generated during pilot period by each physician and (B) average percentage of words changed in artificial intelligence (AI) scribe–generated notes by each physician. Due to technical issues, the percentage of words changed was not recorded for some physicians.

Over the pilot period, a total of 7545 notes were generated using the AI scribe by the 31 physicians in the pilot program. There was substantial variability in the number of notes generated across physicians, with a median (IQR) of 169.0 (42.5-384.5) notes, ranging from 10 to 809 (Figure 1).

Physicians evaluated the quality of 356 (4.7%) of 7545 notes, with a median number of notes evaluated by a physician of 11.0 (IQR 8.5-13.0). Family medicine and internal medicine physicians evaluated most of the notes, 182 (51.1%) and 115 (32.3%), respectively, as these specialties comprised three-quarters (23/31, 74.2%) of the participating physicians. Entering these data for the quality assessments into Qualtrics required a median of only 38.0 (IQR 18.0-96.2) seconds. Physicians usually conducted the quality assessments on the same day as the clinic visit, yielding a median time between the clinic visit and the quality assessment of 3.9 (IQR 1.6-21.1) hours. Among these 356 notes that were scored by physicians, aggregated patient characteristics are summarized in Table S1 in Multimedia Appendix 2, demonstrating a wide representation among patients seen in the health care system.

### Error Frequency and Severity

Among the 356 notes, accidental omissions were the most prevalent quality concern, occurring in 18% (n=64) of the notes, followed by hallucinations (n=41, 11.5%) and accidental inclusions (n=33, 9.3%; Table 2). Bias was only reported in 4 notes (1.1%). In general, quality concerns tied to omissions, hallucinations, or accidental inclusions were rated as mild to moderate (severity ratings of 1‐3). Severity scores of 4 to 5, representing potentially serious or imminent risk of harm if draft errors were not corrected by clinicians, were infrequent—with only 7 (2%) draft notes in this range for accidental omissions, 9 (2.5%) for hallucinations, 5 (1.4%) for accidental inclusions, and 2 (0.6%) for bias.

Finally, the combined note quality score (accounting for severity) was high, with a median quality score of 4.0 (IQR 3.8-4.0). Two-thirds (n=242, 68%) of the notes received a score of 4.0, indicating no quality concerns. A small number of notes (n=11, 3.1%) had an overall score less than 3. Note quality was similar across the specialties (Table 3; Table S2 in Multimedia Appendix 1).

**Table 2.** Summary of concern severity ratings for quality assessments of visit notes (N=356).

Severity score	Inclusion, n (%)	Omission, n (%)	Hallucination, n (%)	Bias, n (%)
5 (severe)	1 (0.3)	3 (0.8)	3 (0.8)	—^a^
4	4 (1.1)	4 (1.1)	6 (1.7)	2 (0.6)
3	9 (2.5)	12 (3.4)	14 (3.9)	1 (0.3)
2	7 (2.0)	17 (4.8)	10 (2.8)	—
1	12 (3.4)	28 (7.9)	8 (2.2)	1 (0.3)
0 (no concerns)	323 (90.7)	292 (82.0)	315 (88.5)	352 (98.9)

a No notes were identified with bias with this severity score.

**Table 3.** Summaries of total scores from the note quality assessments and the percentage of each note edited, as reported by the vendor, by specialty.

Specialty^a^	Total score (n=356)		Percentage edited (n=960)	
	Notes, n (%)	Median (IQR; range)	Notes, n (%)^b^	Median (IQR; range)
Dermatology	11 (3.1)	4.00 (3.50-4.00; 2.60‐4.00)	0 (0)	—^c^
Family practice	182 (51.1)	4.00 (3.80-4.00; 1.80‐4.00)	571 (59.5)	8.3 (2.4-17.3; 0.0‐63.5)
Internal medicine	115 (32.3)	4.00 (3.60-4.00; 1.40‐4.00)	364 (37.9)	9.5 (2.1-25.5; 0.0‐100.0)
Obstetrics and gynecology	4 (1.1)	4.00 (3.80-4.00; 3.20‐4.00)	0 (0)	—
Otolaryngology	26 (7.3)	4.00 (4.00-4.00; 4.00‐4.00)	21 (2.2)	34.4 (21.2-46.8; 8.9‐91.2)
Pediatrics	18 (5.1)	3.80 (3.45-4.00; 3.00‐4.00)	4 (0.4)	22.4 (16.2-33.0; 10.7‐51.7)

a No quality assessments were conducted by the endocrinologist.

b Due to technical difficulties with the data from the vendor, the percentage edited was only obtained at the note level for a portion of all notes and was not available for any notes for dermatology, obstetrics and gynecology, or endocrinology.

c Percentage edited was not available for any artificial intelligence–generated notes for this specialty.

### Percentage Edited

**Figure 2.**
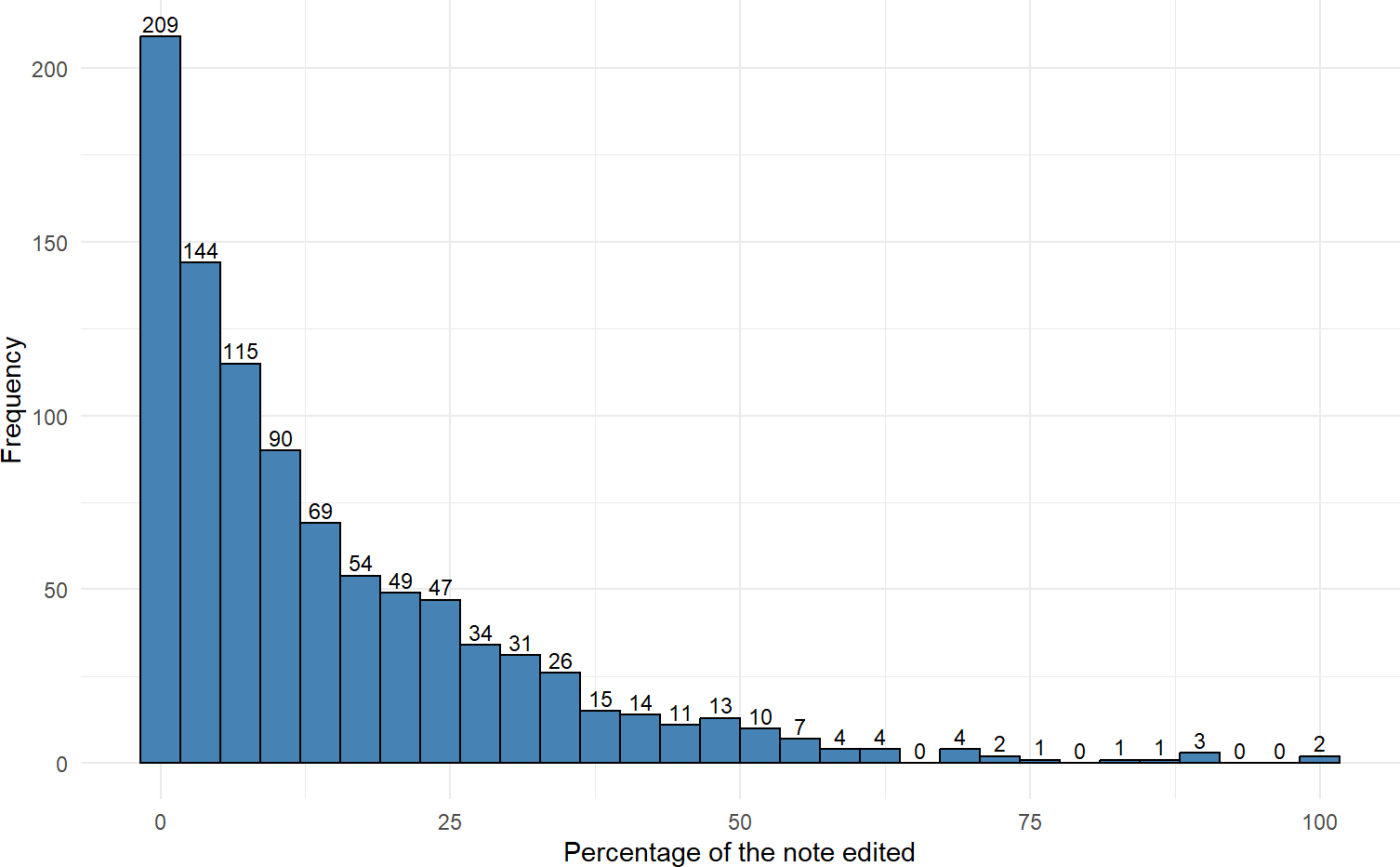
Distribution of percentage of words changed in artificial intelligence–generated clinic visit notes. The x-axis indicates the percentage of the note that was changed, and the y-axis indicates the number of notes with that level of editing.

Of the 7545 AI-created notes, the vendor metric of percentage of words retained was reported for 960 (12.7%) notes. Most of the AI-drafted note content was left unedited by physicians, with a median 9.0% (IQR 2.5%-21.9%; Figure 2) of AI-generated words changed. Notably, 14.9% (143/960) of the AI-generated notes were not edited at all. Individual editing practices varied substantially by physician, with the average percentage of words changed by physician ranging from 1.9% to 69.3% (Figure 1). At the note level, the percentage edited was similar for family practice (median 8.3%, IQR 2.4%-17.3%) and internal medicine (median 9.5%, IQR 2.1%-25.5%) but higher for otolaryngology (median 34.4%, IQR 21.2%-46.8%) and pediatrics (median 22.4%, IQR 16.2%-33.0%; Table 3).

## Discussion

Consistent with other studies, we found that most (337/356, 94.7%) AI scribe platform–generated notes were of high quality and free from significant errors [11,12]. However, accidental omissions were relatively common, identified in 18% (64/356) of the notes evaluated. Most errors were rated as low risk for patient harm; however, 5.3% (19/356) of AI-drafted notes contained an error deemed to pose a risk of serious harm if left uncorrected. Thus, careful clinician review of notes remains imperative.

Because we prioritized patient safety and specifically focused on identifying errors or bias in AI-generated notes, our evaluation tool to capture physician ratings was very brief, allowing rapid assessment of a large number of notes. This contrasts with the longer PDQI-9 instrument that evaluates overall document quality. Natural language processing metrics have also been used to evaluate note quality in previous studies [13], but these metrics do not explicitly identify deviations that could impact patient safety. Our note quality assessment approach is therefore unique in rating the severity of risk posed to patients if the error is not corrected.

Several limitations were encountered in implementing and evaluating this program. First, this was a pilot program, and clinicians volunteered as early adopters of AI scribe technology within the health care system. Second, although the assessment rubric was short, most physicians in the pilot assessed fewer than the requested 20 notes, despite repeated outreach from the project team. Thus, our method of including participants in the pilot and their differential response rates could have biased our results. Third, because physicians reviewed notes from their own clinic visits, identification and rating of the severity of the errors could be biased due to the physician’s individual perspective as well as the passage of time between the visit and note review. However, physicians tended to review notes the same day as the clinic visit, which would reduce recall bias. Finally, to achieve the objectives of our quality improvement project, we did not seek to develop a rigorously validated instrument. Severity ratings were subjective, based on individual physician’s clinical judgment within the context of a particular patient, such that scores could vary among institutions and clinical departments, thus requiring local calibration. Future studies and improvements could include rigorous development of the instrument through multiple reviewer assessments of AI-generated notes relative to recorded transcripts, standardization of severity ratings, and external validation.

In the future, ongoing monitoring of the quality of AI-generated notes will be important to identify changes in the underlying algorithm or practices that could degrade note quality. While our assessment tool is time efficient, it still requires human review, which might impede its use for long-term monitoring. Importantly, we combined direct physicians' ratings with vendor-reported metrics that are more easily obtainable, specifically the percentage of each note that was edited, as a secondary indicator of note quality. Using direct feedback and reporting from end users as well as automatically generated information from platforms will be important for a long-term monitoring approach. Given the occurrence of errors in AI-generated notes, monitoring trends, both in the percentage of notes that are heavily edited or notes that are not edited, will facilitate early identification of changes in physician vigilance in note review. Finally, as our health care system moves toward broader implementation of an AI scribe platform, we are investigating the standardization of quality monitoring by correlating vendor metrics with physician ratings. Given that physician behavior can change over time when adopting AI tools into routine practice, and the performance of the tools themselves may drift, our health system has prioritized long-term monitoring of quality and safety.

In summary, we have outlined a pilot program that might be a generalizable model for other health care systems. Our focused priorities on quality and safety of AI-generated notes, while also maintaining feasibility in the evaluation, were essential for piloting and will likely remain so for broad-scale implementation. Furthermore, we continually engage with the AI vendor to inform their automated metrics that align with our ongoing evaluation priorities. Moving forward, we also plan to implement these approaches and our findings into clinician training in the use of AI technology. With the promise of AI, particularly tools aimed at reducing physician workload and burnout, there is a pathway for rapid deployment while maintaining high standards of patient care.

## Supplementary material

10.2196/86474 Multimedia Appendix 1 Note quality survey user interface and error types by specialty.

10.2196/86474 Multimedia Appendix 2 Patient demographics.

## Abbreviations

AI: artificial intelligence
PDQI-9: Physician Documentation Quality Instrument
UCDH: University of California, Davis Health
