# Supplementary material for: Quality of Clinical Notes Created by Ambient Listening Generative AI: Pragmatic Prospective Pilot Study
Source: JMIR Med Inform. 2026 Apr 17;14:e86474. doi: 10.2196/86474 (PMC13089619; doi:10.2196/86474)
Supplement: Multimedia Appendix 1 [file medinform-v14-e86474-s001.docx]

| 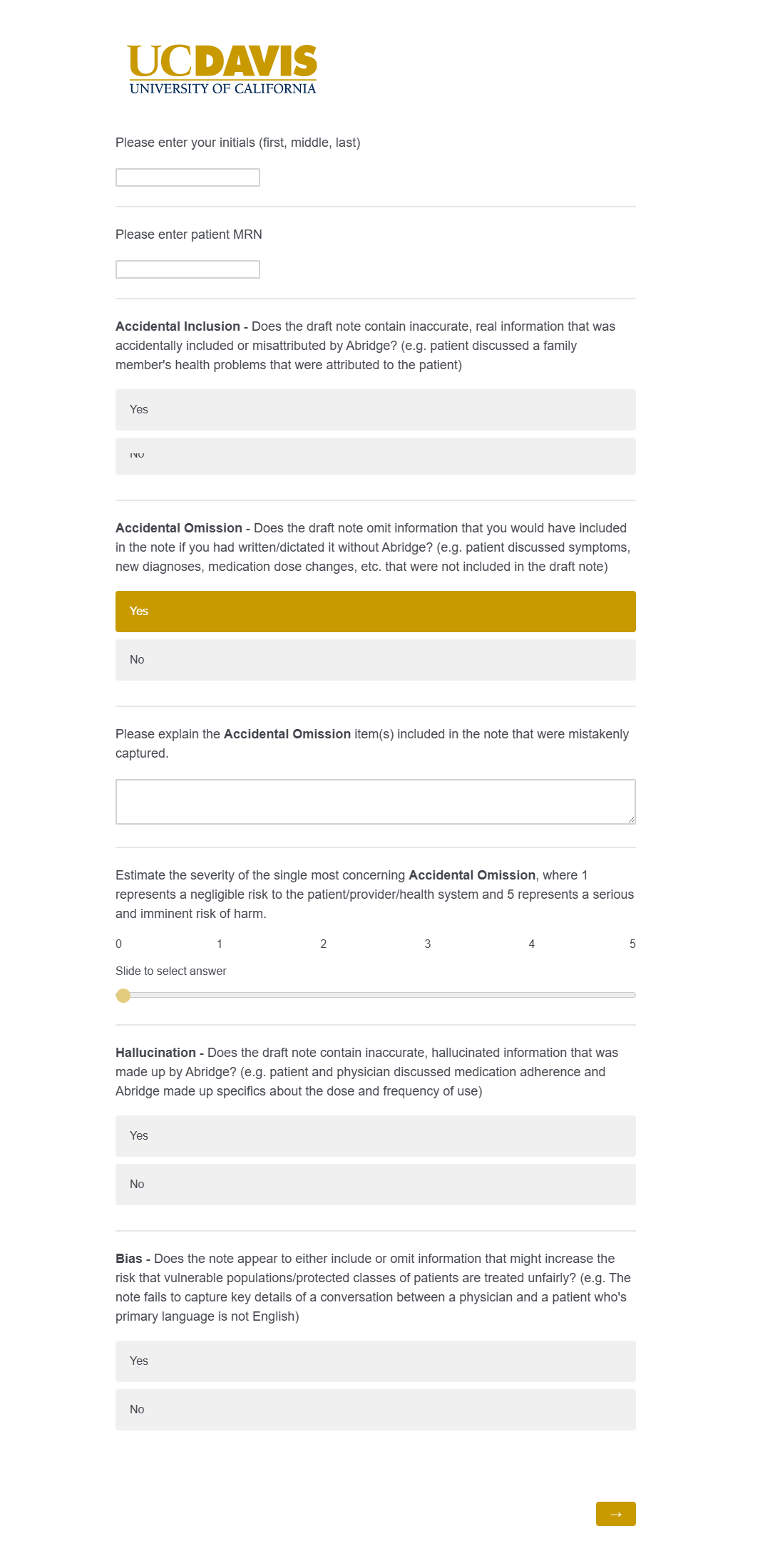 | 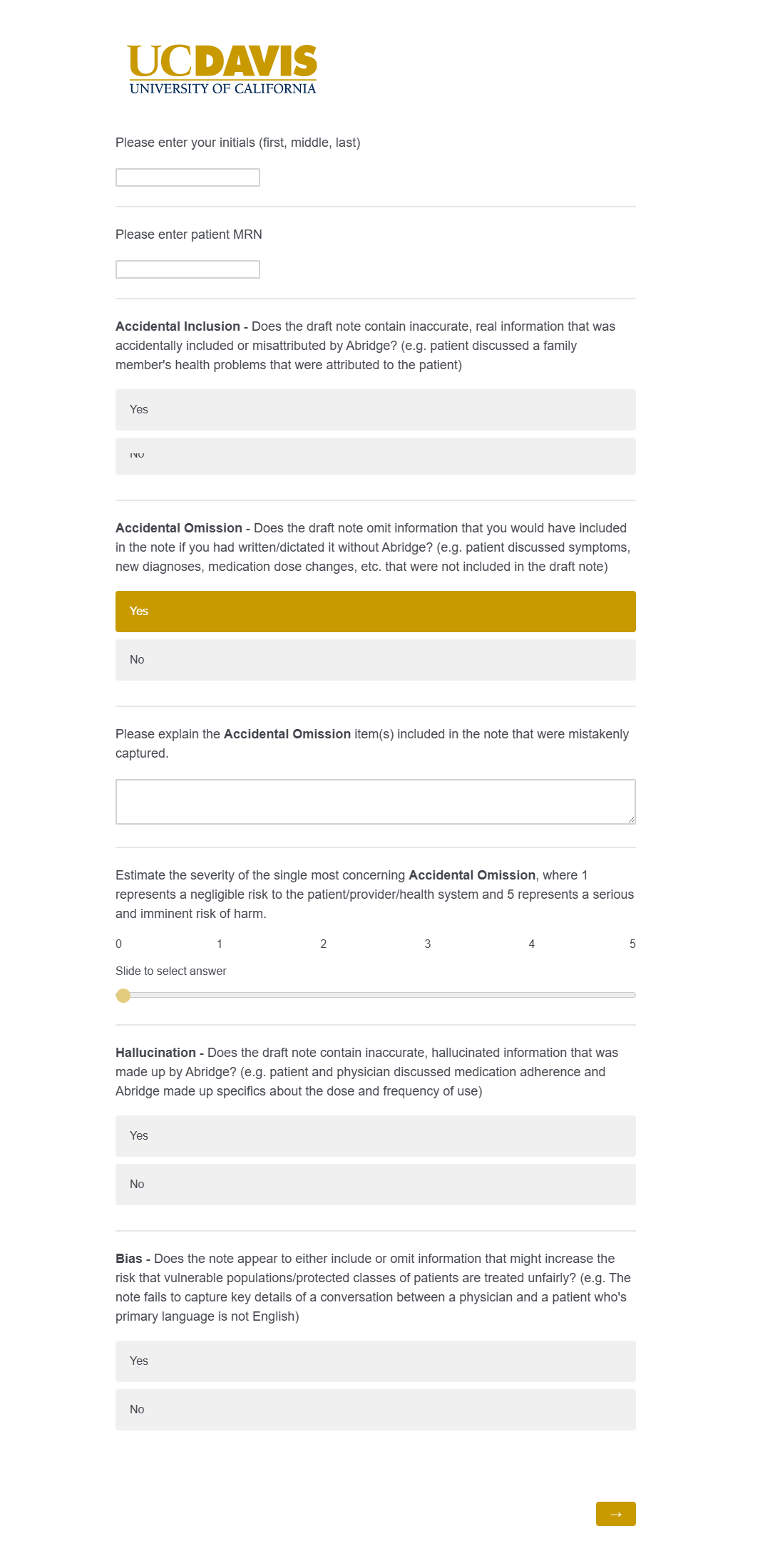 |
| --- | --- |

Figure S1. Note quality survey user-interface. The survey uses branching logic. If the user responds “Yes” to a particular error type, a slider is revealed for the user to rate the error’s severity as shown for Accidental Omission.

| Table S2. Severity of each error type by physician specialty. | | | | | | | |
| --- | --- | --- | --- | --- | --- | --- | --- |
| **N physicians** | **Dermatology (N=1)** | **Family Practice (N=12)** | **Internal Medicine (N=11)** | **Obstetrics/Gyn (N=1)** | **Otolaryngology (N=3)** | **Pediatrics (N=2)** | **Total (N=30^a^)** |
| **Number of notes** | 11 | 182 | 115 | 4 | 26 | 19 | 356 |
| **Accidental Inclusion** |  |  |  |  |  |  |  |
| 5 | 0 (0.0%) | 0 (0.0%) | 1 (0.9%) | 0 (0.0%) | 0 (0.0%) | 0 (0.0%) | 1 (0.3%) |
| 4 | 1 (9.1%) | 2 (1.1%) | 1 (0.9%) | 0 (0.0%) | 0 (0.0%) | 0 (0.0%) | 4 (1.1%) |
| 3 | 0 (0.0%) | 2 (1.1%) | 5 (4.3%) | 0 (0.0%) | 0 (0.0%) | 2 (11.1%) | 9 (2.5%) |
| 2 | 0 (0.0%) | 1 (0.5%) | 4 (3.5%) | 1 (25.0%) | 0 (0.0%) | 1 (5.6%) | 7 (2.0%) |
| 1 | 0 (0.0%) | 6 (3.3%) | 4 (3.5%) | 0 (0.0%) | 0 (0.0%) | 2 (11.1%) | 12 (3.4%) |
| 0 | 10 (90.9%) | 171 (94.0%) | 100 (87.0%) | 3 (75.0%) | 26 (100.0%) | 13 (72.2%) | 323 (90.7%) |
| **Accidental Omission** |  |  |  |  |  |  |  |
| 5 | 0 (0.0%) | 2 (1.1%) | 1 (0.9%) | 0 (0.0%) | 0 (0.0%) | 0 (0.0%) | 3 (0.8%) |
| 4 | 0 (0.0%) | 2 (1.1%) | 2 (1.7%) | 0 (0.0%) | 0 (0.0%) | 0 (0.0%) | 4 (1.1%) |
| 3 | 0 (0.0%) | 7 (3.8%) | 4 (3.5%) | 0 (0.0%) | 0 (0.0%) | 1 (5.6%) | 12 (3.4%) |
| 2 | 0 (0.0%) | 8 (4.4%) | 8 (7.0%) | 0 (0.0%) | 0 (0.0%) | 1 (5.6%) | 17 (4.8%) |
| 1 | 0 (0.0%) | 13 (7.1%) | 11 (9.6%) | 0 (0.0%) | 0 (0.0%) | 4 (22.2%) | 28 (7.9%) |
| 0 | 11 (100.0%) | 150 (82.4%) | 89 (77.4%) | 4 (100.0%) | 26 (100.0%) | 12 (66.7%) | 292 (82.0%) |
| **Hallucination** |  |  |  |  |  |  |  |
| 5 | 0 (0.0%) | 0 (0.0%) | 3 (2.6%) | 0 (0.0%) | 0 (0.0%) | 0 (0.0%) | 3 (0.8%) |
| 4 | 0 (0.0%) | 2 (1.1%) | 4 (3.5%) | 0 (0.0%) | 0 (0.0%) | 0 (0.0%) | 6 (1.7%) |
| 3 | 3 (27.3%) | 5 (2.7%) | 5 (4.3%) | 0 (0.0%) | 0 (0.0%) | 1 (5.6%) | 14 (3.9%) |
| 2 | 1 (9.1%) | 4 (2.2%) | 3 (2.6%) | 1 (25.0%) | 0 (0.0%) | 1 (5.6%) | 10 (2.8%) |
| 1 | 0 (0.0%) | 5 (2.7%) | 2 (1.7%) | 0 (0.0%) | 0 (0.0%) | 1 (5.6%) | 8 (2.2%) |
| 0 | 7 (63.6%) | 166 (91.2%) | 98 (85.2%) | 3 (75.0%) | 26 (100.0%) | 15 (83.3%) | 315 (88.5%) |
| **Bias** |  |  |  |  |  |  |  |
| 5 | 0 (0.0%) | 0 (0.0%) | 0 (0.0%) | 0 (0.0%) | 0 (0.0%) | 0 (0.0%) | 0 (0.0%) |
| 4 | 0 (0.0%) | 0 (0.0%) | 2 (1.7%) | 0 (0.0%) | 0 (0.0%) | 0 (0.0%) | 2 (0.6%) |
| 3 | 0 (0.0%) | 0 (0.0%) | 1 (0.9%) | 0 (0.0%) | 0 (0.0%) | 0 (0.0%) | 1 (0.3%) |
| 2 | 0 (0.0%) | 0 (0.0%) | 0 (0.0%) | 0 (0.0%) | 0 (0.0%) | 0 (0.0%) | 0 (0.0%) |
| 1 | 0 (0.0%) | 1 (0.5%) | 0 (0.0%) | 0 (0.0%) | 0 (0.0%) | 0 (0.0%) | 1 (0.3%) |
| 0 | 11 (100.0%) | 181 (99.5%) | 112 (97.4%) | 4 (100.0%) | 26 (100.0%) | 18 (100.0%) | 352 (98.9%) |
| ^a^ The endocrinologist participating in the pilot did not evaluate note quality during the pilot. | | | | | | | |
