# Supplementary material for: Quality of Clinical Notes Created by Ambient Listening Generative AI: Pragmatic Prospective Pilot Study
Source: JMIR Med Inform. 2026 Apr 17;14:e86474. doi: 10.2196/86474 (PMC13089619; doi:10.2196/86474)
Supplement: Multimedia Appendix 2 [file medinform-v14-e86474-s002.docx]

| Table S1. Demographics of patients for clinical notes assessed | |
| --- | --- |
| **Attribute** | **Overall (N=335)** |
| **Sex** |  |
| Female | 185 (55.2%) |
| **Race/Ethnicity** |  |
| American Indian or Alaska Native | 1 (0.3%) |
| Asian | 24 (7.2%) |
| Black or African American | 16 (4.8%) |
| Latinx / Hispanic | 41 (12.2%) |
| Multi-Racial | 13 (3.9%) |
| Native Hawaiian or Other Pacific Islander | 2 (0.6%) |
| Other | 16 (4.8%) |
| Unknown / Declined | 20 (6.0%) |
| White / Caucasian | 202 (60.3%) |
| **Age** |  |
| Mean (SD) | 56.70 (21.20) |
| **Preferred Language** |  |
| Arabic | 1 (0.3%) |
| Chinese (Traditional) | 1 (0.3%) |
| Decline to State | 2 (0.6%) |
| English | 321 (95.8%) |
| Hindi | 1 (0.3%) |
| Russian | 2 (0.6%) |
| Samoan | 1 (0.3%) |
| Spanish | 3 (0.9%) |
| Unspecified | 3 (0.9%) |
| **CA HPI Quartile^a^** |  |
| Missing | 25 |
| 1 | 19 (6.1%) |
| 2 | 57 (18.4%) |
| 3 | 123 (39.7%) |
| 4 | 111 (35.8%) |
| **ADI State Rank^b^** |  |
| Missing | 23 |
| 1 | 3 (1.0%) |
| 2 | 9 (2.9%) |
| 3 | 31 (9.9%) |
| 4 | 22 (7.1%) |
| 5 | 51 (16.3%) |
| 6 | 45 (14.4%) |
| 7 | 46 (14.7%) |
| 8 | 55 (17.6%) |
| 9 | 38 (12.2%) |
| 10 | 12 (3.8%) |
| ^a^ CA HPI = California Healthy Places Index  ^b^ ADI = Area Deprivation Index | |
